# Fecal microbiome transplant from patients with lactation mastitis promotes mastitis in conventional lactating mice

**DOI:** 10.3389/fmicb.2023.1123444

**Published:** 2023-04-14

**Authors:** Chao-Yue Kong, Yi-Qin Yang, Bing Han, Hui-Ling Chen, Yu-Qin Mao, Jia-Ting Huang, Li-Shun Wang, Zhan-Ming Li

**Affiliations:** ^1^Center for Traditional Chinese Medicine and Gut Microbiota, Minhang Hospital, Fudan University, Shanghai, China; ^2^Institute of Fudan-Minhang Academic Health System, Minhang Hospital, Fudan University, Shanghai, China; ^3^Traditional Chinese Medicine Department, Minhang Hospital, Fudan University, Shanghai, China

**Keywords:** lactation mastitis, fecal microbiota transplantation, pro-inflammatory, microbiome, mammary gland

## Abstract

**Introduction:**

Lactation mastitis seriously severely affects the health of lactating females and their infants, yet the underlying causes of clinical lactation mastitis remain unclear.

**Methods:**

In this study, we used microbiota-humanized mice as a model to investigate the role of gut microbiota in lactation mastitis. We compared the fecal microbiota of lactation mastitis patients and healthy individuals and conducted fecal microbiota transplantation (FMT) experiments in an antibiotic-pretreated mouse model to test whether gut microbes contribute to human lactation mastitis.

**Results:**

Our results showed that gut microbiota diversity was reduced and dysbiosis was present in lactating mastitis patients. FMT from lactation mastitis patients (M-FMT), but not from healthy individuals (H-FMT), to antibiotic-treated mice resulted in lactation mastitis. The inflammation in mice caused by gut microbiota from lactating mastitis patients appears to be pervasive, as hepatocytes from mice that received feces from lactating mastitis patients showed marked swelling. In addition, serum pro-inflammatory factors, including IL-4, IL-17, MPO, IL-6, IL-1β, and TNF-α, were significantly increased in the M-FMT group. The Firmicutes/Bacteroidetes ratio (F/B), a biomarker of gut dysbiosis, was significantly increased in the M-FMT group. At the phylum level, Actinobacteria were significantly increased, and Verrucomicrobia were significantly decreased in the M-FMT group. At the genus level, *Ruminococcus* and *Faecalibacterium* were significantly reduced, while *Parabacteroides* were significantly increased in the feces of both patients with lactation mastitis and M-FMT mice. Moreover, our study revealed an “amplification effect” on microbiota differences and mastitis disease following human-to-mouse FMT.

**Conclusion:**

Collectively, our findings demonstrate that the gut microbiota in lactating mastitis patients is dysbiotic and contributes to the pathogenesis of mastitis.

## Introduction

Lactation mastitis is a common inflammatory reaction of mammary gland tissue, most common in the first 3 months of breastfeeding, affecting almost all lactating mammals (Omaleki et al., 2016). Lactation mastitis has brought significant health and social burdens to humans. Women with lactation mastitis suffer from localized breast pain, redness, swelling, warmth, and systemic symptoms (Jimenez et al., 2015). The incidence of lactation mastitis is about 2–33%. Most women with lactation mastitis have been prescribed antibiotics (e.g., dicloxacillin and cephalexin), which increases the risk of weaning (Barbosa-Cesnik et al., 2003; Spencer, 2008).

Although the aetiology of lactation mastitis has been studied for decades, it is not fully understood whether there is a specific pathogen behind it, and evidence from various studies points in different directions (Peters, 2004). Dairys are most susceptible to pathogens such as *Staphylococcus aureus* and coagulase-negative staphylococci during milk production (Osterman and Rahm, 2000). Microorganisms opportunistically invade and colonize the mammary gland, causing an immediate inflammatory response (Barbosa-Cesnik et al., 2003; Goldstone et al., 2016). As currently identified pathogens (such as *S. aureus*) are usually present in the nasal cavity, skin, hair, and throat mucosa, skin flora may be one of the risk factors for lactation mastitis during breastfeeding (Foxman et al., 2002; Barbosa-Cesnik et al., 2003).

At the same time, a large body of research argues against the identification of specific pathogens as a single cause of lactation mastitis. In recent years, gut microbiota has received more and more attention. Numerous studies have shown intestinal flora plays a vital role in improving disease and maintaining health (Lynch and Pedersen, 2016; Ma et al., 2018; Hoque et al., 2022). The gut microbiota has been implicated in the progression of various diseases, such as cancer, diabetes, non-alcoholic fatty liver disease, obesity, and hypertension (Louis et al., 2014; Kong et al., 2019, 2021). It has been shown that mice with disturbed gut microbiota (antibiotic treatment) increase the incidence of *S. aureus*-induced mastitis in mice (Hu et al., 2020). Additionally, the fecal microbiota of mastitis cows causes mastitis-like symptoms in germ-free mice (Ma et al., 2018; Hoque et al., 2022). The above research results show that gut microbiota plays a vital function in the progression of mastitis. Several studies have reported that humanized rodent models have significantly contributed to understanding human physiology and pathophysiology (Weingarden and Vaughn, 2017; Chen et al., 2020; Hu et al., 2020). To date, the aetiology of clinical lactation mastitis remains controversial. At the same time, no studies reported gut microbiota characteristics in lactation mastitis patients. We speculate that disturbed gut microbiota may cause lactation mastitis in clinical patients. In this study, we sequenced the gut microbiota in stool samples from patients with lactation mastitis and identified a critical role for the gut microbiome in the induction of lactation mastitis.

## Materials and methods

### Study subjects

Seventeen females with lactation mastitis and eighteen healthy lactating females were enrolled in this study from May 2019 to October 2021 in the Department of Breast specialist clinic of the Minhang Hospital, China. The lactation mastitis group was 25 to 37 years old, with a median age of 31.2 ± 3.5 years. The healthy group was 26 to 36 years old, with a median age of 30.2 ± 3.4 years. Volunteers with lactation mastitis and healthy lactating females were recruited during acute mastitis. Before sample collection, the volunteers had not received any antibiotics or drug treatment. The diagnostic criteria for acute lactation mastitis are clinical symptoms (breast pain, chills, myalgias, and fever) and signs of inflammation (Fernandez et al., 2014). The clinical manifestations are breast pain, poor milk discharge, and local breast lumps, wedge-shaped or irregular in shape, and can occur in any part of the breast. The breast skin may appear red, swollen, hot, and painful, and the skin temperature in the lesion area may increase and cause tenderness (Barbosa-Cesnik et al., 2003). Venous blood was drawn from volunteers, and the white corpuscle count, the percentage of neutrophils, lymphocytes, and the level of C-reactive protein (CRP) were detected and recorded. Fresh stool samples were collected from lactating women with lactating mastitis and healthy lactating women. Fresh fecal samples collected were immediately for fecal microbiota transplantation (FMT) or stored in 20% glycerol for freezing. Any procedures of this research protocol were carried out after being approved by the Ethics Committee of Minhang Hospital (Shanghai, China).

### Animals and experimental protocol

We randomly selected five patients with lactation mastitis and five healthy controls as faecal donors for FMT. According to our previous research (Li et al., 2023), on the day of FMT, 200 mg stool samples from the donor were mixed and suspended in twice the fecal volume of PBS, vortexed and filtered by the sterile filter. The recipient mice were gavaged with 200 μl of the fecal suspension once a day through the experiment.

Based on previous research on mastitis (Zhao et al., 2023a,b), female and male mice (7–8 weeks of age) were housed in a specific pathogen-free (SPF) environment on a 12-h light and 12-h bright cycle. Mice were gavaged with antibiotics (vancomycin, 100 mg/kg; neomycin sulfate, metronidazole, and ampicillin, 200 mg/kg) once daily for five consecutive days. After antibiotic treatment, mice were inoculated with the prepared fecal contents by gavage daily for three consecutive days. Then keep it twice a week for 62 days. After six weeks of gavage of the fecal microbiota mixture, mice were housed in a 2–3:1 ratio of males to females overnight, and pregnancy was confirmed by examining the presence of vaginal sperm. Mice fed with the microbiota of lactation mastitis and healthy patients were termed M-FMT and H-FMT, respectively.

### Histopathological analysis

Mammary gland, liver, and colon tissues were collected from lactating females with mastitis. The mammary gland tissues were immediately fixed in 10% formalin and then stained with hematoxylin and eosin (H&E staining) for examination under a light microscope. Immunohistochemistry analysis was performed on the mammary gland tissues using markers MPO and CD45. Fix the breast tissue samples in 10% neutral buffered formalin and process them routinely to obtain tissue sections embedded in 4 μm paraffin. Then, stain the sections with hematoxylin and eosin and examine them under a light microscope. According to the literature (Camperio et al., 2017), use a semi-quantitative scoring system based on the features of inflammatory cell infiltration to measure the observed lesions, especially those characterized by the accumulation of polymorphonuclear leukocytes. The grades from 1 to 4 apply to the following changes: 0, no damage, no interstitial and/or alveolar inflammatory cell infiltration, and undamaged tissue; 1, focal to multifocal, mild interstitial and/or alveolar inflammatory cell infiltration, and undamaged tissue; 2, multifocal, moderate interstitial and/or alveolar inflammatory cell infiltration, and undamaged tissue; 3, severe, diffuse interstitial and/or alveolar inflammatory cell infiltration and focal tissue damage; 4, severe, diffuse interstitial and/or alveolar inflammatory cell infiltration, and extensive areas of necrosis. A score of 1 to 3 represented mild to moderate inflammation and tissue damage.

### Inflammatory cytokines assay

The four inflammatory cytokines (Interleukin-4 (IL-4), Interleukin-6 (IL-6), Interleukin-1β (IL-1β), Interleukin-17 (IL-17), and Tumor Necrosis Factor-α (TNF-α)) and one infection biomarker (Myeloperoxidase (MPO)) were evaluated using an ELISA assay kit according to the manufacturer’s instructions (Abcam, United Kingdom).

### 16S rRNA gene sequencing and analysis of feces

According to the manufacturer, fecal DNA was extracted from approximately 250 mg specimen using the QIAamp Fast DNA Stool Mini Kit (QIAGEN, Hilden, Germany). The 16S rRNA gene was amplified by PCR with universal bacterial primers (F: CCTAYGGGRBGCASCAG R: GGACTACNNGGGTATCTAAT) targeting the V3 and V4 region. Before pooling the libraries, barcoded PCR products were purified using the QIAGEN Gel Extraction Kit (Axygen, United States) and quantified using the FTC-3000™ Real-Time PCR (Shanghai Fenglin Medical Laboratory). PCR products from different tissue samples were indexed, mixed in equal proportions, and sequenced on the Illumina NovaSeq-PE250 platform of Personalbio Bio-Tech (Shanghai) Co., Ltd. Based on OTU information, microbial α- and β-diversity was analyzed by bioinformatics. Venn diagrams can count the number of common and unique OTUs in multiple samples and visually represent the resemblance overlap and the difference in OTU composition in fecal samples (Fouts et al., 2012). Based on the sequencing data, the number of taxa contained in each of the seven taxonomic levels of the domain, phylum, class, order, family, genus, and species in the species annotation results of these samples was counted (Oberauner et al., 2013). To further analyze the differentially rich taxa responsible for the classification between the two groups, the unsupervised Random Forest taxonomic analysis method was used (McKinney et al., 2006). Finally, the relationship between differential flora and lactation mastitis was analyzed by Spearman correlation.

### Statistical analysis

Data were represented as mean ± standard error of the mean (SEM) and were analyzed using an unpaired two-tailed student’s *t*-test. Statistical analysis was performed with GraphPad Prism 8.0 software (GraphPad Software, San Diego, United States). The results are considered statistically significant at *p* < 0.05, *p* < 0.01 or *p* < 0.001.

## Results

### Disturbed intestinal flora in patients with lactation mastitis

**Figure 1:**
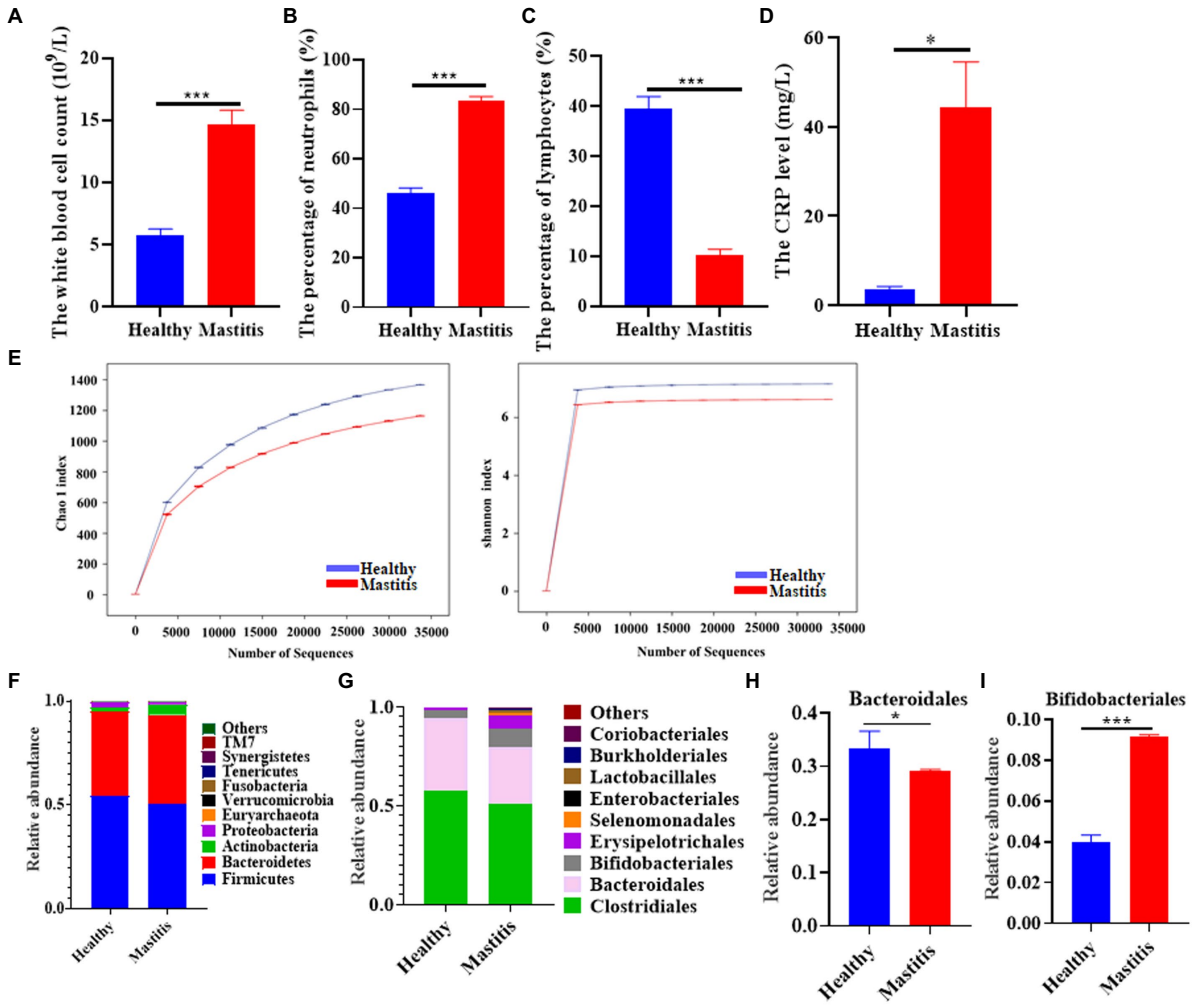
The pattern of immune response markers and intestinal flora in healthy females and patients with lactation mastitis. **(A)** The number of white corpuscles, **(B)** the percentage of neutrophils and **(C)** lymphocytes, and the level of **(D)** CRP were counted. **(E)** Alpha diversity, including Chao 1 and Shannon diversity index. **(F)** shows the gut microbiota composition in terms of relative abundance at the phylum level. **(G)** shows the gut microbiota composition in terms of relative abundance at the order level. **(H,I)** Relative abundances of bacteroidales and Bifidobacteriales between healthy and mastitis. The data represent the means ±SEMs (*n* = 8–9 person per group). *p < 0.05, ***p < 0.001 by *t*-test.

A total of 18 healthy lactating females and 17 lactation mastitis females were included in the present study. Blood test results showed that the white blood cell count, percentage of neutrophils, and CRP content in the lactation mastitis group (mastitis) were significantly higher than in the healthy group (healthy). In addition, the percentage of lymphocytes in the lactation mastitis group was significantly reduced compared to the healthy group (Figures 1A–D). The gut microbiome of the two groups was further investigated. Alpha diversity indices, including Chao1 and Shannon indices, slightly decreased in the lactation mastitis group (Figure 1E). Consistently, the Venn diagram showed that the number of OTUs in the healthy group was 15,431, which was about 26% higher than that in the mastitis group (Supplement Figure S1A). Consistently, the Venn diagram showed that the healthy group had a number of OTUs of 15,431, approximately 26% higher than the lactation mastitis group. According to PCoA, the gut microbiota of the lactation mastitis group differed significantly from that of the healthy group using the unweighted UniFrac distance (Supplement Figure S1B). The study found that with the further deepening of the classification tree, from phylum to family to genus, there were significant differences between the two groups (Supplement Figure S1C). Next, we analyzed the relative abundance of gut microbiota. We observed that four phyla (Firmicutes, Bacteroidota, Actinobacteria, and Proteobacteria) and four order (Clostridiales, Bacteroidales, Bifidobacteriales, and Erysipelotrichales) dominate the gut microbiota communities in both healthy and mastitis group (Figures 1F,G). The relative abundance of Bacteroidales was significantly lower in the mastitis group compared with the healthy (Figure 1H). In addition, the relative abundance of Bifidobacteriales was significantly higher in the mastitis group compared with the healthy group (Figure 1I).

### Lactation mastitis phenotype was transferable by fecal transplantation

To further explore whether disturbed gut microbiota contributes to the progression of lactation mastitis, we transferred fecal microbiota from lactation mastitis patients to antibiotic-treated mice.

**Figure 2:**
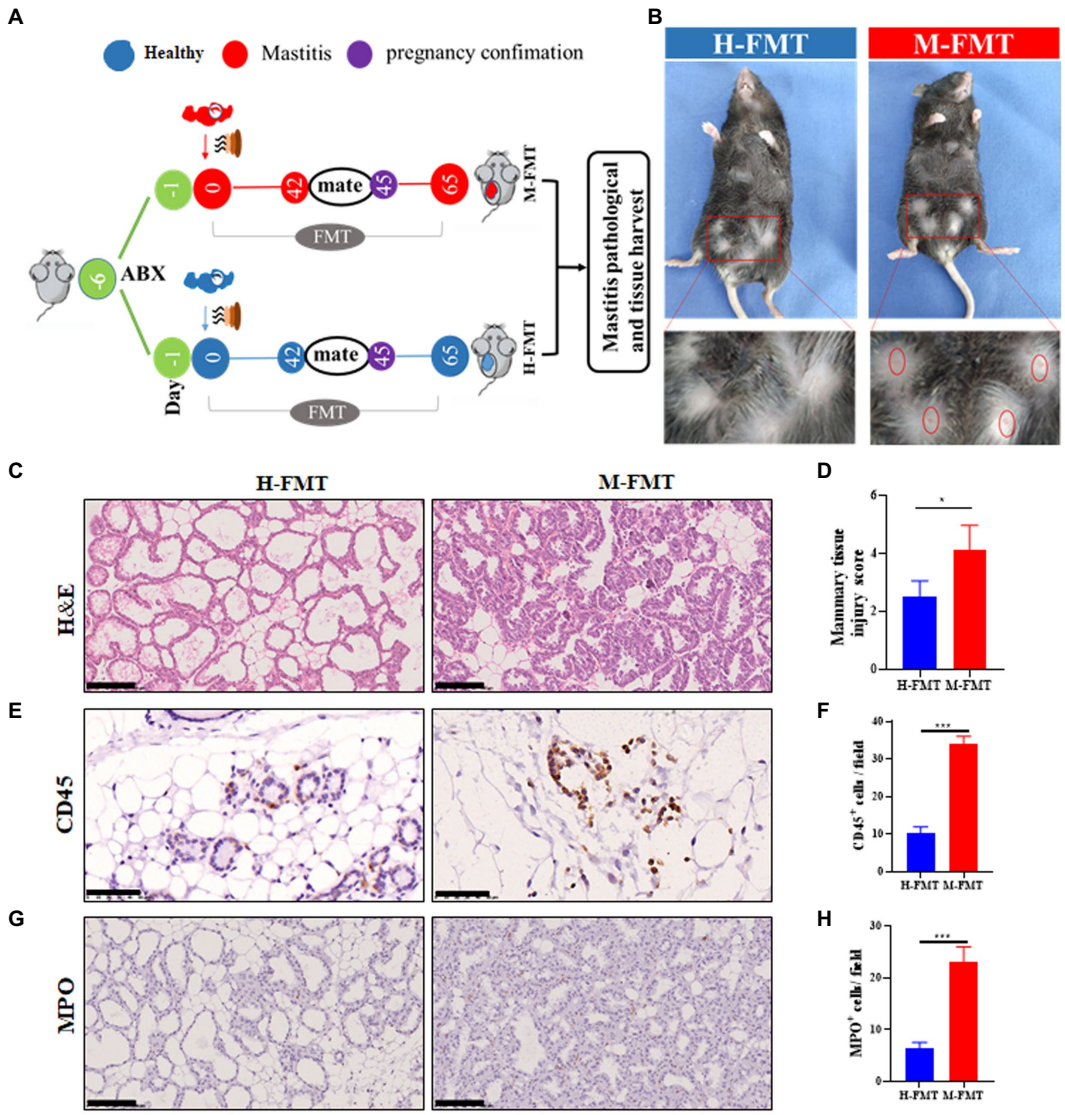
Administration of fecal bacteria from patients with lactation mastitis to mice induced a series of mastitis-like phenotypes. **(A)** Schematic representation of FMT. Fresh fecal from five healthy and five lactation mastitis donors were mixed and used as a single source for H-FMT and M-FMT mice. Following antibiotic treatment, the recipient mice were randomly divided and orally inoculated daily for three consecutive days and two times per week for 62 days with prepared fecal contents. **(B)** Pathological changes in mammary gland surface, where two abdominal mammary glands were swelling in the mastitis group of mice on day 65 after FMT. The breast of the mice was highlighted by a red frame. **(C)** Representative photomicrographs of hematoxylin–eosin (H&E) stained mammary tissue. **(D)** The injury score of mammary glands (*n* = 8–10 mice per group). **(E)** CD45 immunohistochemical staining sections at ×400 magnification. **(F)** A bar graph showing the number of CD45^+^ cells in the H-FMT and M-FMT mice (*n* = 8–10 mice per group). **(G)** MPO immunohistochemical staining sections at ×400 magnification. **(H)** A bar graph showing the number of MPO^+^ cells in the H-FMT and M-FMT mice. The data represent the means ±SEMs (*n* = 8–10 mice per group). *p < 0.05, ***p < 0.001 by *t*-test. Scale bar, 100 μm.

After the mice were treated with antibiotics for 5 consecutive days, the mice received fecal samples from normal lactating females or females with lactation mastitis (Figure 2A). Comparing the two groups after FMT, it was found that the inflammatory reaction of the mammary glands in mice fed with feces of lactation mastitis was more severe than in the mice fed with healthy women. The surface of the mammary glands of the mice in the M-FMT (mice fed with fecal bacteria from lactation mastitis females) group was red and swollen, and obvious symptoms of mastitis appeared. No obvious pathological changes were observed in the H-FMT (mice fed with fecal bacteria from healthy lactating females) group (Figure 2B). Under H&E staining, the M-FMT group showed ruptured mammary lobules, damaged mammary acinus, and epithelial cell destruction (Figures 2C,D). However, in the H-FMT group, the mammary lobules were intact, the mammary acinus was intact, and there were no obvious pathological changes.

Consistent with the results of H&E staining, mammary glands CD45 and MPO-positive cells were also significantly increased in the mastitis group. (Figures 2E–H). As the first type of protein tyrosine phosphatase receptor, CD45 is expressed on all nucleated hematopoietic cells and plays a central role in adaptive immunity (Penninger et al., 2001). The results of CD45 immunohistochemistry were shown in Figure 2E. Compared with H-FMT, M-FMT significantly increased the distribution of MPO in breast tissue (Figures 2E,F). MPO is a heme protease containing a heme prosthetic group secreted by neutrophils, monocytes, and macrophages in certain tissues and is a member of the heme peroxidase superfamily. The results of MPO immunohistochemistry were shown in Figure 2G. Compared with H-FMT, M-FMT significantly increased the distribution of MPO in breast tissue (Figures 2G,H).

### Fecal microbiota from lactation mastitis patients induced a systemic inflammatory response in donor mice

**Figure 3:**
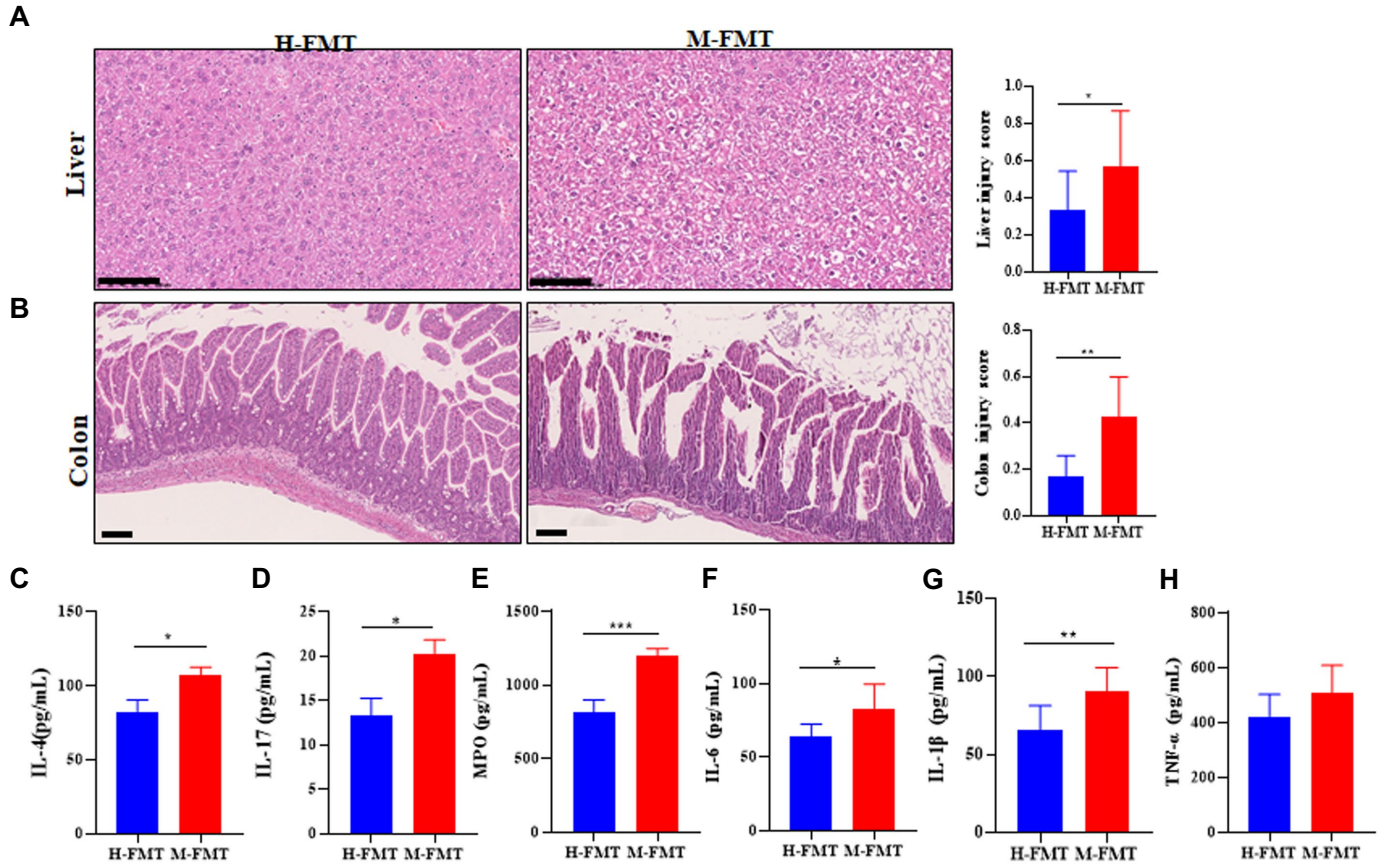
The murine inflammation induced by lactation mastitis patient’s intestinal microbiota seemed pervasive. **(A)** Representative photomicrographs of the hematoxylin–eosin-stained liver (×200) (left). The injury score of the liver (right). **(B)** Representative photomicrographs of hematoxylin–eosin-stained colon (×100) (left). The injury score of colons (right). Quantification of inflammatory cytokines including IL-4 **(C)**, IL-17 **(D)**, MPO **(E)**, IL-6 **(F)**, IL-1β **(G)**, and TNF-α **(H)** in serum using ELISA. The assays were all performed for the two groups of mice on Day 65 after FMT. The data represent the means ±SEMs (*n* = 8–10 mice per group). *p < 0.05, **p < 0.01, ***p < 0.001 by *t*-test. Scale bar, 100 μm.

The inflammatory response caused by the gut microbiota in lactation mastitis patients is almost systemic. H&E staining of mouse liver sections showed blurring, hyperemia, and hepatocyte ballooning degeneration in the M-FMT group compared with the H-FMT group (Figure 3A). In addition, fecal microbiota transplantation (FMT) from patients with lactation mastitis but not healthy lactating female into antibiotic-treated mice resulted in colon inflammation (Figure 3B). The pathological sections of the colons of the mice in the M-FMT group showed severe disturbance of the mucosal structure (epithelial cell necrosis, extended subepithelial spaces, and enlarged sub-epithelial spaces). In contrast, mice in the H-FMT group exhibited normal intestinal mucosa and intact villi (Figure 3B). Further, we analyzed the levels of serum inflammatory cytokines. The results showed that compared with the H-FMT, the serum levels of IL-4 (Figure 3C), IL-17 (Figure 3D), MPO (Figure 3E), IL-6 (Figure 3F), IL-1β (Figure 3G) and TNF-α (Figure 3H) were increased in the M-FMT group.

### Differences in gut microbiota between healthy and lactation mastitis mice after FMT

**Figure 4:**
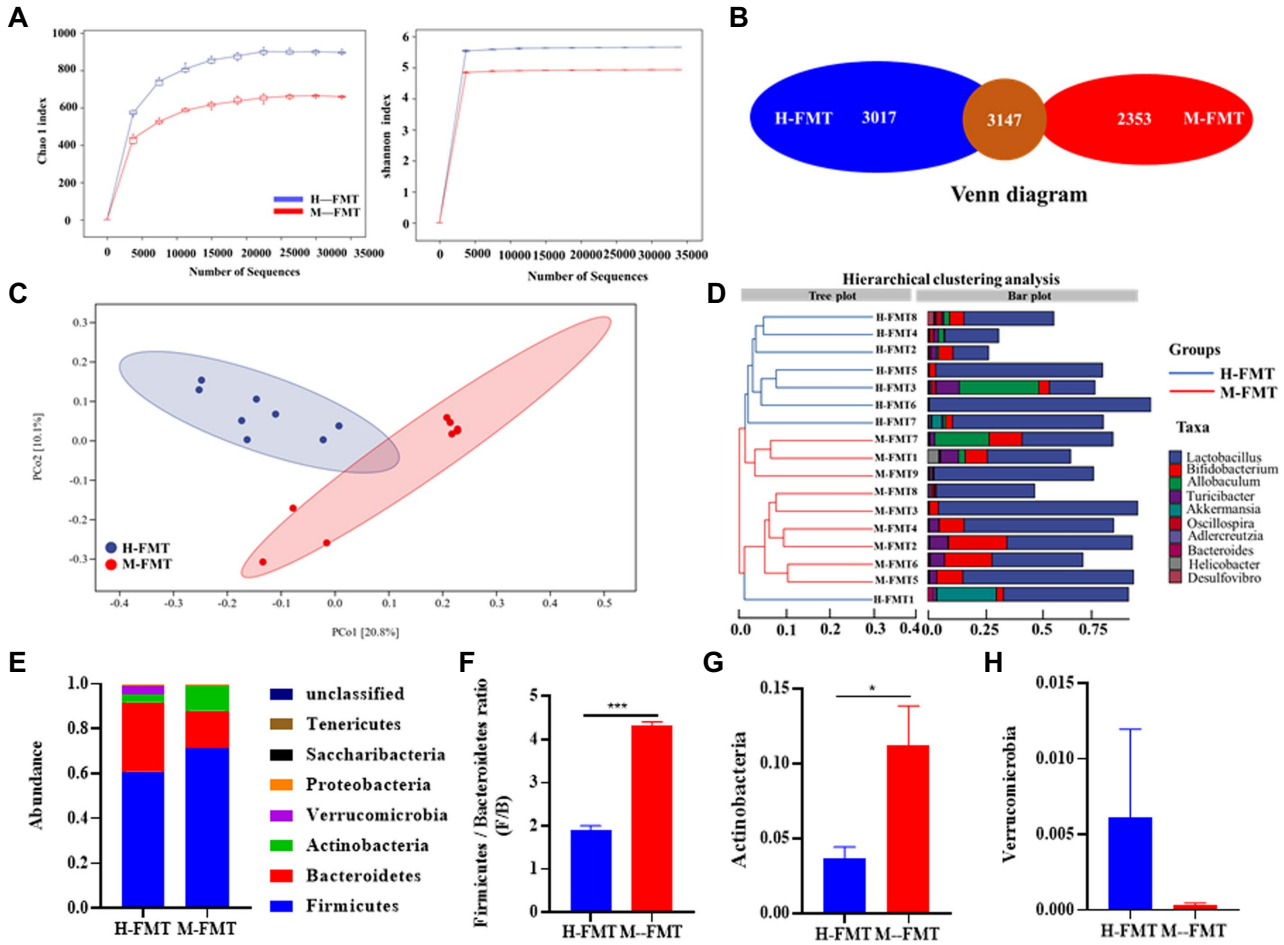
The distinction between healthy and lactation mastitis intestinal microbiota in the mice after FMT. **(A)** Alpha diversity, including Chao 1 and Shannon diversity index. **(B)** Venn diagram. **(C)** Unweighted UniFrac distances between healthy and lactation mastitis groups in a PCoA. **(D)** Hierarchical clustering tree on genus level using the Bray–Curtis distance (left)and the component proportion of bacteria at the genus level in each group (right). **(E)** Relative abundance of the phyla in the feces. **(F)** The Firmicutes: bacteroidetes ratio(F/B) was calculated as a biomarker for gut dysbiosis. **(G,H)** Relative abundances of Actinobacteria and Verrucomicrobia between H-FMT and M-FMT groups. *n* = 8–10 mice per group. *p < 0.05, ***p < 0.001 by *t*-test.

To explore the mechanism of differential outcome between the two groups of mice after FMT, 16S rRNA gene amplicons in the feces of each mouse were analyzed on day 65 after FMT. Compared with H-FMT, the Alpha diversity index (including Chao1 and Shannon diversity index) decreased slightly in the M-FMT group (Figure 4A). Consistently, the Venn diagram showed that the number of OTUs in the H-FMT group was 3,017, which was about 22% higher than that in the M-FMT group (Figure 4B). Figure 4C shows a PCoA map of the gut microbiome in mice, using the unweighted UniFrac distance of the samples, showing that the gut microbiome of the M-FMT and H-FMT groups was significantly different. Hierarchical clustering tree using unweighted UniFrac distances (left) and component proportions of each group of the bacterial genus (right) at the OTU level. Consistently, the hierarchical clustering tree showed that the genus-level abundances of the H-FMT group and M-FMT group were quite different, and most of them were clustered together in the same group (Figure 4D). The overall gut microbiota profile of the two groups was further assessed at the phylum level. A total of 7 phyla were identified. Significant differences were observed in Firmicutes, Bacteroidetes, Proteobacteria, and Verrucomicrobia (Figure 4E). Moreover, M-FMT intervention reduced the increase in Firmicutes/Bacteroidetes ratio (F/B) compared to H-FMT (Figures 4E,F), which has been reported to be the hallmark of disease-driven dysbiosis (Cotillard et al., 2013; Yasir et al., 2015). A significant increase in Actinobacteria was observed in the M-FMT group, along with a significant decrease in Verrucomicrobia (Figures 4G,H).

### Human fecal microbiota transplant amplified disease effects in mice

**Figure 5:**
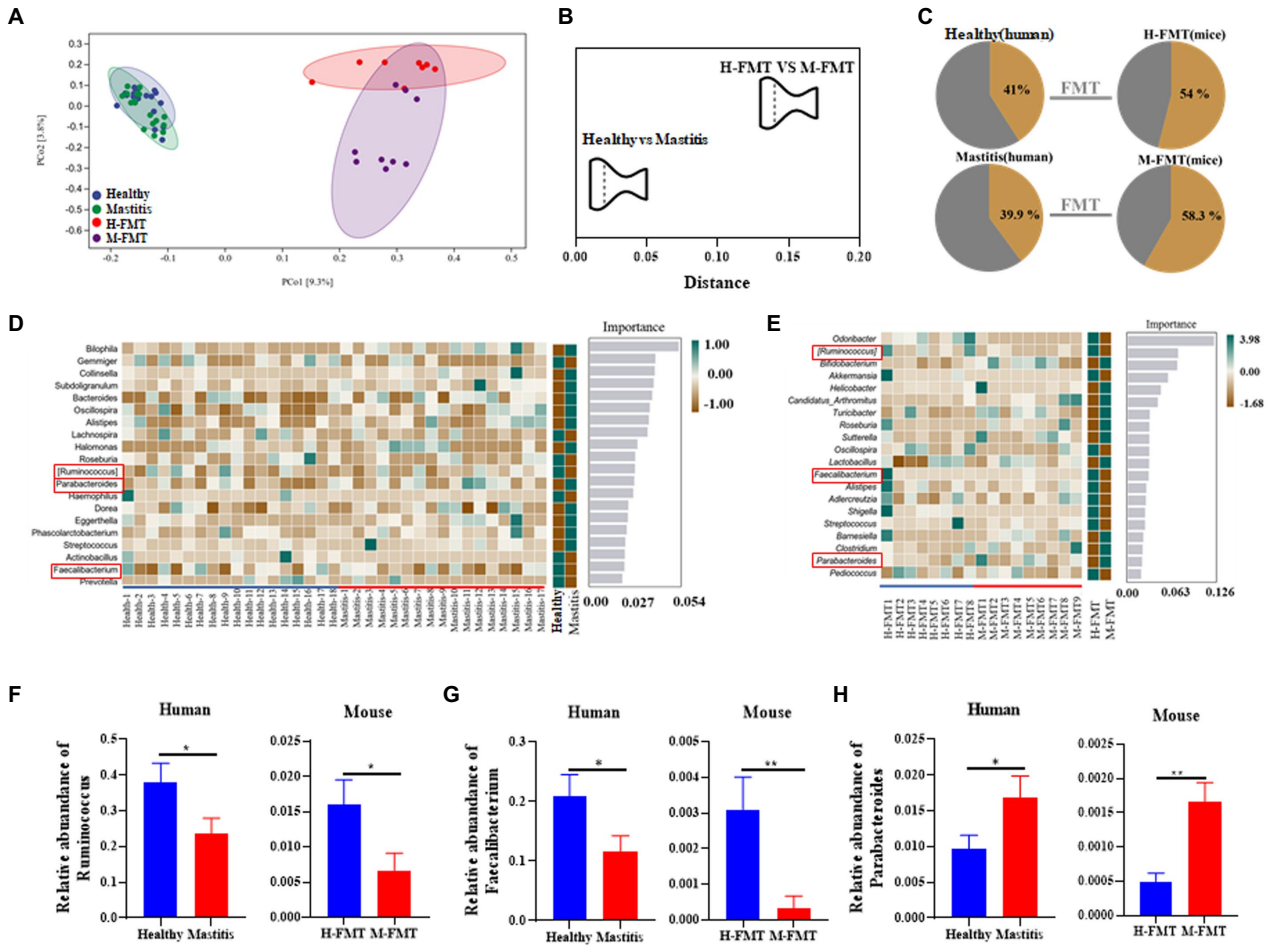
Comparison of mastitis-associated microbiota in humans and those in the mouse. PCoA clustering **(A)** and relative similarity **(B)** of person and mice microbiota based on organismal structure (*via* Meta-Storm distance) were shown. **(C)** Unique and shared OTUs before and after FMT in person and mice. **(D)** Random Forest (RF) classification of genus-level gut microbiota in two groups of volunteers. **(E)** Random Forest (RF) classification of gut microbiota in two groups of recipient mice. **(F–H)** Relative abundances of *Ruminococcus*, *Faecalibacterium*, and *Parabacteroides* between the human and mouse groups. *n* = 8–15 person or mice per group. *p < 0.05 by *t*-test.

Previous results showed that transplantation of fecal microbiota from patients to antibiotic-treated mice led to lactation mastitis, suggesting that the gut microbiota is the cause, not the consequence, of lactation mastitis (Figure 2). On this basis, it was also found that cross-species mammalian fecal bacterial colonization not only led to the disease’s recurrence but also expanded its effect. As demonstrated by a 7-fold magnification of the difference (OTU) between healthy and lactation mastitis microbiota (averaged distance of 0.02 between groups healthy and mastitis in female volunteers versus 0.14 in mice; Figures 5A,B). We next probed how such “amplification effect” occurred and why the vast difference between donor microbiota and xenomicrobiota ended up with similar disease outcomes. In the gut microbiota of the murine recipients, most of the genus-level taxa (54% for healthy pairs and 58.3% for mastitis pairs) were from those of the female volunteer donors (Figure 5C). Random forest (RF) classification was performed on the genus level of gut microbes in volunteers and recipient mice, respectively (Figures 5D,E).

**Figure 6:**
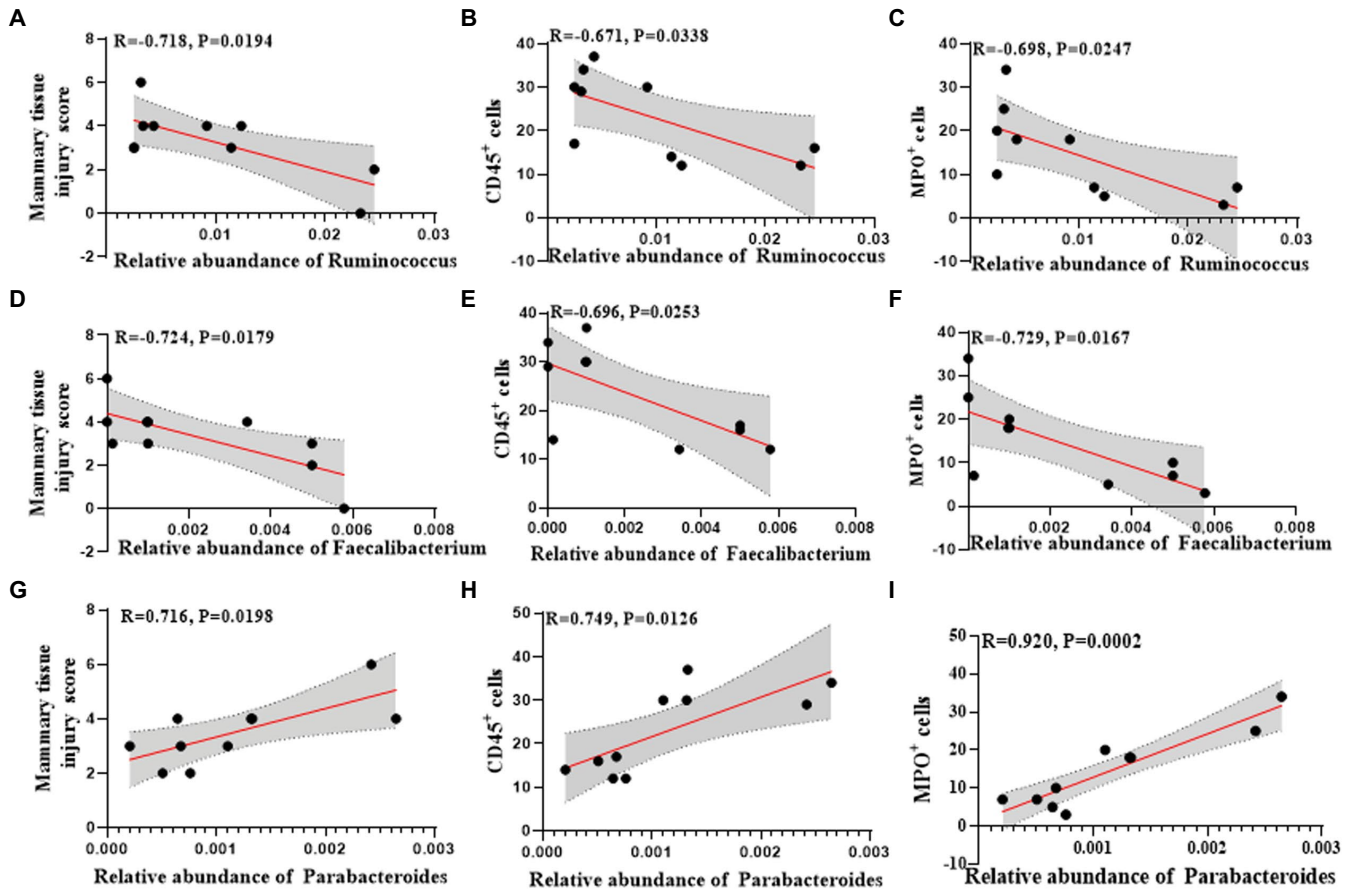
Univariate linear regression between *Ruminococcus*, *Faecalibacterium* and *Parabacteroides* relative abundance in fecal samples and mammary tissue injury score, CD45^+^ cells and MPO^+^ cells of the matched mice. **(A)** Spearman correlation between *Ruminococcus* and mammary tissue injury score. **(B)** Spearman correlation between *Ruminococcus* and CD45^+^cells. **(C)** Spearman correlation between *Ruminococcus* and MPO^+^ cells. **(D)** Spearman correlation between *Faecalibacterium* and mammary tissue injury score. **(E)** Spearman correlation between *Faecalibacterium* and CD45^+^cells. **(F)** Spearman correlation between *Faecalibacterium* and MPO^+^ cells. **(G)** Spearman correlation between *Parabacteroides* and mammary tissue injury score. **(H)** Spearman correlation between *Parabacteroides* and CD45^+^ cells. **(I)** Spearman correlation between *Parabacteroides* and MPO^+^ cells.

Among them, the changes of *Ruminococcus*, *Parabacteroides* and *Faecalibacterium* at the genus level were consistent in humans and mice. *Ruminococcus* and *Faecalibacterium* were significantly reduced in the mastitis group compared to the healthy group (Figures 5F,G). In accordance, the change of *Ruminococcus* was negatively associated with the mammary tissue injury score (*R* = −0.718, *p* = 0.0194), CD45^+^ cells (*R* = −0.671, *p* = 0.0338), and MPO^+^ cells (*R* = −0.698, *p* = 0.0247) (Figures 6A–C). Furthermore, *Parabacteroides* was elevated in the mastitis group compared to the healthy group (Figure 5H). In accordance, the change of *Faecalibacterium* was positively associated with the mammary tissue injury score (*R* = −0.724, *p* = 0.0179), CD45^+^ cells (*R* = −0.696, *p* = 0.0253), and MPO^+^ cells (*R* = −0.729, *p* = 0.0167) (Figures 6D–F). In addition， the change of *Parabacteroides* was negatively associated with the mammary tissue injury score (*R* = 0.716, *p* = 0.0198), CD45^+^ cells (*R* = 0.749, *p* = 0.0126), and MPO^+^ cells (*R* = 0.920, *p* = 0.0002) (Figures 6G–I).

### The correlation between differential flora and disease indicators in lactation mastitis population

**Figure 7:**
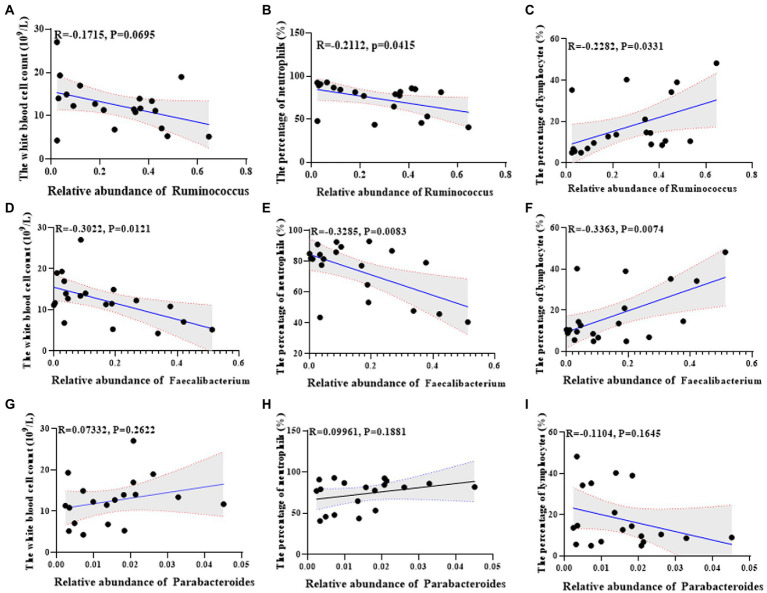
Univariate linear regression between *Ruminococcus*, *Faecalibacterium* and *Parabacteroides* relative abundance in fecal samples and the white blood cell count, the percentage of neutrophils and the percentage of lymphocytes of the matched mice. **(A)** Spearman correlation between *Ruminococcus* and the white blood cell count. **(B)** Spearman correlation between *Ruminococcus* and the percentage of neutrophils. **(C)** Spearman correlation between *Ruminococcus* and the percentage of lymphocyte cells. **(D)** Spearman correlation between *Faecalibacterium* and the white blood cell count. **(E)** Spearman correlation between *Faecalibacterium* and the percentage of neutrophils. **(F)** Spearman correlation between *Faecalibacterium* and the percentage of lymphocyte cells. **(G)** Spearman correlation between *Parabacteroides* and the white blood cell count. **(H)** Spearman correlation between *Parabacteroides* and the percentage of neutrophils. **(I)** Spearman correlation between *Parabacteroides* and the percentage of lymphocyte cells.

Further, we analyzed the correlation between the found differential flora and serum indexes of the lactation mastitis population. The change of *Ruminococcus* was negatively related to the white blood cell count (*R* = −0.1715, *p* = 0.0695) and the percentage of neutrophils (*R* = −0.2112, *p* = 0.0415) (Figures 7A,B). The change of *Ruminococcus* was positively related to the percentage of lymphocytes (*R* = 0.2282, *p* = 0.0331) (Figure 7C). The change of *Faecalibacterium* was negatively associated with the white blood cell count (*R* = −0.332, *p* = 0.0121), the percentage of neutrophils (*R* = −0.3285, *p* = 0.0083) (Figures 7D,E). The change of *Faecalibacterium* was positively related to the percentage of lymphocytes (*R* = 0.3363, *p* = 0.0074) (Figure 7F). The change of *Parabacteroides* was positively related to the white blood cell count (*R* = 0.07332, *p* = 0.2622), the percentage of neutrophils (*R* = 0.09961, *p* = 0.1881) (Figures 7G,H). The change of *Parabacteroides* was related to the percentage of lymphocytes (*R* = −0.1104, *p* = 0.1645) (Figure 7I).

## Discussion

Clinically, mastitis usually occurs during lactation, which refers to the acute suppurative inflammation of the mammary gland, and it is more common in women who are breastfeeding after childbirth, especially in primiparas. It often occurs three to four weeks after childbirth. Because the etiology of lactation mastitis is unknown, methods for predicting, preventing, and treating the disease are unclear. The study wanted to look for possible clues in the gut microbiota. The study is the first to examine intestinal flora in people with lactation mastitis. The results showed that the gut microbiome was disrupted in lactation mastitis patients compared with healthy lactating women. When the feces of people with lactation mastitis were given to antibiotic-treated mice by gavage, the mice developed mastitis-like symptoms with systemic inflammation. It is suggested that the disturbance of intestinal flora may be the cause of lactation mastitis in patients. The gut dysbiosis in lactation mastitis observed in our study was obvious.

The present study found that *Parabacteroides* were significantly lower in patients with lactation mastitis. Also, similar results were shown in mice. Preliminary studies have shown that *Parabacteroides* induce depression in mice with Crohn’s disease (Gomez-Nguyen et al., 2021). We observed dysbiosis in the gut microbiota, such as increased abundance of *Ruminococcus* and *Faecalibacterium*, in lactating female volunteers with lactation mastitis (Figure 5). This is consistent with previous reports observed in mastitis cows (Ma et al., 2018). Although our study cannot rule out that a single strain may cause mastitis, it is still worth looking forward to the future use of gut microbiota to prevent and treat lactation mastitis. *Ruminococcus* is a common anaerobic gram-positive intestinal bacterium. *Ruminococcus* was inversely associated with proinflammatory cytokines in mastitis cows (Chuang et al., 2021). This is consistent with the increased abundance of *Ruminococcus* in healthy populations than in mastitis in this study. It is suggested that *Ruminococcus* may be a potential strain to improve mastitis in the human population.

*Faecalibacterium* is ubiquitous in the human gut and is a promising microorganism for developing next-generation probiotics or biotherapeutics. *Faecalibacterium* is ubiquitous in the human intestinal tract and is one of the important producers of butyric acid. *Faecalibacterium* is a promising microorganism with an anti-inflammatory strain that protects the gut from pathogens and can be used to develop next-generation probiotics or biopharmaceuticals (Heinken et al., 2014; Riviere et al., 2016). Previous studies have shown that *Faecalibacterium* improves or may be helpful in Alzheimer’s treatment and reduces fat accumulation in high-fat-induced obese mice (Munukka et al., 2017). In the present study, *Faecalibacterium* was significantly reduced in both lactation mastitis patients and mice, suggesting that the anti-inflammatory effect was diminished, thus causing inflammation in the mice.

In human studies, probiotics have been shown to have similar effects to antibiotics (Klostermann et al., 2008; Arroyo et al., 2010) while avoiding the negative consequences of antibiotics. At the same time, antibiotic treatment is also a common and routine treatment strategy for lactation mastitis (Jahanfar et al., 2016). However, it may lead to residual antibiotics in milk endangering neonatal health while disrupting normal microbiota development and digestion in breastfed infants: tract and respiratory tract. The results of this study provide a reference for future microbial prevention and treatment of human lactation mastitis. Furthermore, mouse FMT amplifies the role of human mastitis-associated gut microbes, suggesting that humanized mouse models provide some help in screening and localization of microbiota.

## Conclusion

We characterized the gut microbiota of lactation mastitis patients. Furthermore, through the fecal bacteria transplantation experiment, it was confirmed that the gut microbiota may be a critical factor in the induction of lactation mastitis. Targeting the gut microbiota may be considered a novel approach for future interventions in lactational mastitis.

## Data availability statement

The datasets presented in this study can be found in online repositories. The names of the repository/repositories and accession number(s) can be found at: https://www.ncbi.nlm.nih.gov/bioproject/PRJNA874851.

## Ethics statement

The studies involving human participants were reviewed and approved by Ethics Committee of Minhang Hospital. The patients/participants provided their written informed consent to participate in this study. The animal study was reviewed and approved by Ethics Committee of Minhang Hospital. Written informed consent was obtained from the individual(s) for the publication of any potentially identifiable images or data included in this article.

## Author contributions

C-YK and Y-QY analyzed the data and performed the statistical analysis. Y-QY collected patient information and stool samples. C-YK and Z-ML wrote the manuscript. C-YK, H-LC, Y-QM, J-TH, L-SW, and BH conducted the research. Z-ML designed the research and analyzed the data. All authors contributed to the article and approved the submitted version.

## Funding

This work is supported financially by grants from the National Natural Science Foundation of China (81803601 and 81872245), Fundamental Research Funds for Shanghai Municipal Health Commission (20214Y0327, 20214Y0328 and 2022YQ052), Fundamental Research Funds for Minhang Hospital (2021MHJC01, 2021MHJC02, 2022MHBJ01, and 2023MHBJ01), Shanghai Sailing Program (Grant No. 23YF1438700), and Open Research Fund of State Key Laboratory of Genetic Engineering, Fudan University (SKLGE-2112).

## Conflict of interest

The authors declare that the research was conducted in the absence of any commercial or financial relationships that could be construed as a potential conflict of interest.

## Publisher’s note

All claims expressed in this article are solely those of the authors and do not necessarily represent those of their affiliated organizations, or those of the publisher, the editors and the reviewers. Any product that may be evaluated in this article, or claim that may be made by its manufacturer, is not guaranteed or endorsed by the publisher.

## Supplementary material

The Supplementary material for this article can be found online at: https://www.frontiersin.org/articles/10.3389/fmicb.2023.1123444/full#supplementary-material
