# Supplementary figures and images for: Fecal microbiome transplant from patients with lactation mastitis promotes mastitis in conventional lactating mice
Source: Front Microbiol. 2023 Apr 14;14:1123444. doi: 10.3389/fmicb.2023.1123444 (PMC10140588; doi:10.3389/fmicb.2023.1123444)

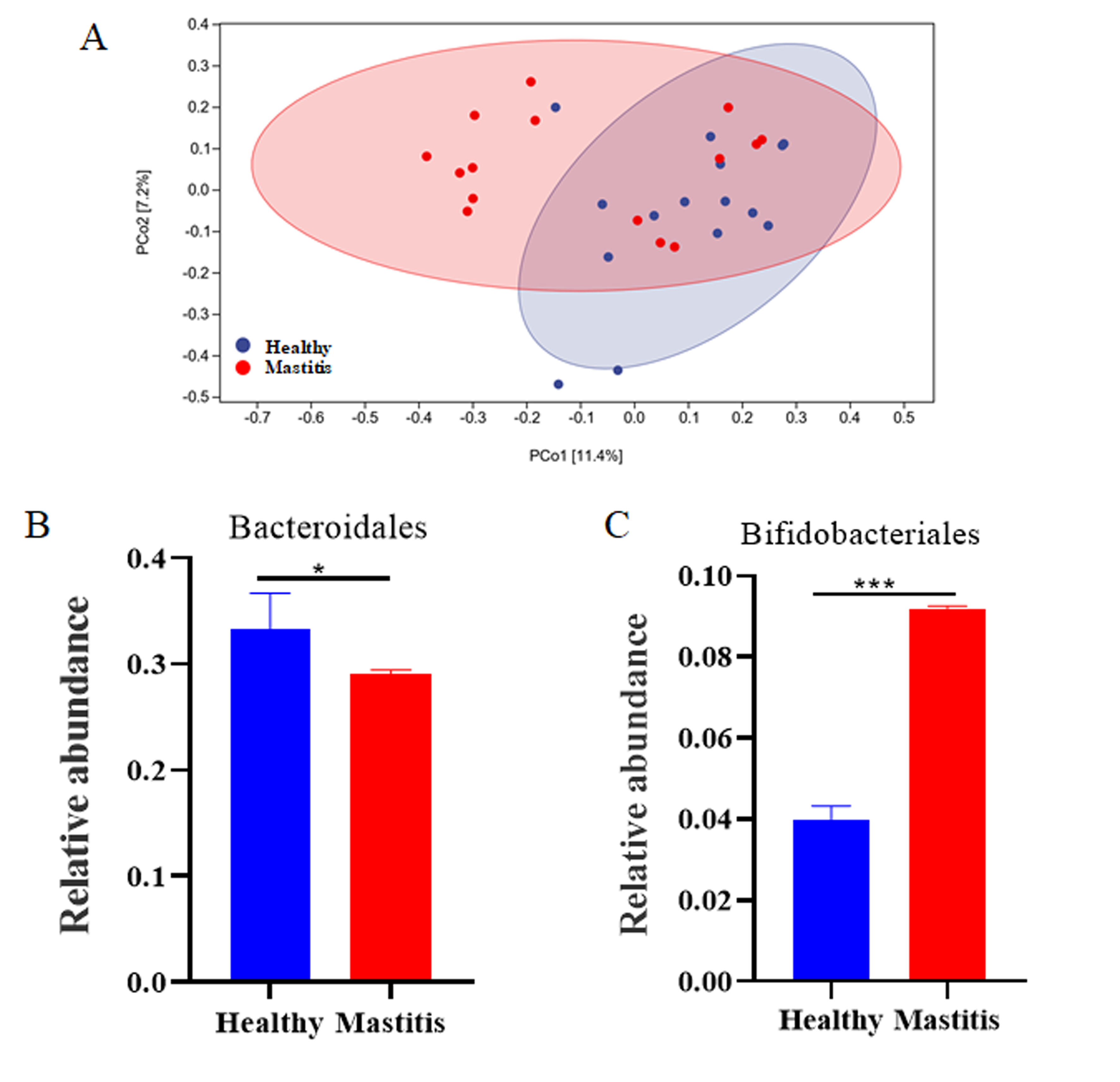

Supplement: Supplementary file 1 [file Image_1.TIF]
